# Assessment of the impact of the COVID-19 pandemic on the productivity of teaching hospitals in Brazil

**DOI:** 10.1590/S2237-96222024v33e20231176.en

**Published:** 2024-12-06

**Authors:** Maria Stella de Castro Lobo, Marcos Pereira Estellita Lins, Henrique de Castro Rodrigues, Gabriel Martins Soares

**Affiliations:** 1 Universidade Federal do Rio de Janeiro, Instituto de Estudos em Saúde Coletiva, Rio de Janeiro, RJ, Brazil; 2 Universidade Federal do Estado do Rio de Janeiro, Escola de Engenharia de Produção, Rio de Janeiro, RJ, Brazil; 3 Universidade Federal do Rio de Janeiro, Instituto Alberto Luiz Coimbra de Pós-Graduação e Pesquisa de Engenharia, Rio de Janeiro, RJ, Brazil; 4 Hospital Universitário Clementino Fraga Filho, Serviço de Epidemiologia e Avaliação e Laboratório de Informação para Gestão Hospitalar, Rio de Janeiro, RJ, Brazil

**Keywords:** Hospitales docentes, Investigación Operativa, Benchmarking, Eficiencia Organizacional, Covid-19, Teaching Hospitals, Operations Research, Benchmarking, Organizational Efficiency, COVID-19

## Abstract

**Objectives:**

To analyze the influence of the COVID-19 pandemic on the productivity of general teaching hospitals in Brazil, by region and legal entity, and to propose parameters of care.

**Methods:**

This was an observational study by means of mathematical modeling with data envelopment analysis and Malmquist index, using data on inputs and healthcare output before (2019) and during (2021) the pandemic.

**Results:**

A total of 149 general teaching hospitals were analyzed, 32 of which were considered efficient. There was a decrease in productivity across all regions and legal entity. To bring all inefficient hospitals to the efficiency frontier generated by the model, there is a need to increase output by 2,205,856 (96.5%) hospitalizations and 872,264 (107.4%) surgeries.

**Conclusion:**

The decline in hospital productivity resulted from the social commitment of hospitals during the pandemic, with a change in the care delivery pattern. The mathematical model used allows for the generation of parameters to facilitate the efficient recovery of care services after the end of public health emergency, and can be applied to hospital planning.

## Study contributions

**Main results:**

The COVID-19 pandemic led to a reduction in productivity of general teaching hospitals in Brazil across all regions and in all legal entity types. In order to recover, these hospitals will need to increase their average hospitalizations by 96.5% and surgeries by 107.4%.

**Implications for services:**

The study presents a methodology that can be adapted and replicated in the management of healthcare services in the country, by defining an efficiency score and calculating the parameters, in a scenario of care recovery following the end of the public health emergency.

**Perspectives:**

Additional qualitative analysis and application of DEA- Malmquist in subsequent years will validate dynamic planning, which considers multiple fluctuations and the influence of new factors and contexts that impact productivity (such as the pandemic).

## RESUMEN

**Objetivos:**

Analizar la influencia de la pandemia de COVID-19 en la productividad de hospitales generales docentes de Brasil, por región y naturaleza jurídica, y proponer parámetros asistenciales.

**Métodos:**

Estudio observacional mediante modelamiento matemático con análisis envolvente de datos e índice de Malmquist, utilizando datos de recursos y producción asistencial antes (2019) y durante (2021) la pandemia.

**Resultados:**

Se analizaron 149 hospitales docentes, 32 se consideraron eficientes. Hubo una baja productividad en todas las regiones y naturalezas jurídicas. Para que todos los hospitales ineficientes alcancen la frontera de eficiencia generada por el modelamiento, es necesario aumentar la producción en 2.205.856 (96,5%) hospitalizaciones y 872.264 (107,4%) cirugías.

**Conclusión:**

La caída de la productividad hospitalaria fue consecuencia del compromiso social de los hospitales durante la pandemia, con cambios en el estándar de atención. El modelo matemático utilizado permite generar parámetros para una recuperación eficiente de atención y puede aplicarse a planificación hospitalaria.

## INTRODUCTION

Coping with the COVID-19 pandemic required a joint effort from society and national public health. Federal, state and municipal health resources were pooled for the hiring of healthcare professionals and acquisition of medical equipment, such as oxygen, sedatives and personal protective equipmen^t^.^1^ Given the high transmissibility and potential severity in the first two years of the pandemic, there was a 47.0% increase in intensive care unit (ICU) beds and a 4.7% increase in other beds, in addition to the establishment of field hospitals and the reconfiguration of units for exclusive care for people with COVID-1^9.1^,^2^

Teaching hospitals (THs) played a role in this process with different strategies, such as suspension of outpatient appointments and elective surgeries, the expansion of intensive care beds, reinforcement of biosafety protocols, the hiring of new professionals, training of healthcare teams, suspension of teaching activities and the development of research on the topi^c^.^3^

It is worth noting that the adoption of pandemic response measures meant that treatment for other diseases was foregon^e^,^4^ leading to changes in the profile of hospital admissions and productive efficiency. Productivity is defined as the ratio between the volume of outputs provided and inputs used by the same productive unit. Technical efficiency is measured by comparing the productivity of similar units, in order to assess the maximum production potential regarding the available input^s^.^5^ There are different methods for analyzing productivity, including the least squares method , total factor productivity, stochastic frontier analysis and data envelopment analysis *–* DE^A^.^5^

DEA is a linear programming technique that measures the performance of productive units, Known as decision-making unit (DMU) , which consume multiple inputs – such as beds, equipment and human resources – to generate various outputs, such as hospitalizations, surgeries and consultation^s^.^6^ mathematical programming is usually used to evaluate a collection of possible alternative courses of action en route to selecting one which is best. In this capacity, mathematical programming serves as a planning aid to management. Data Envelopment Analysis reverses this role and employs mathematical programming to obtain ex post facto evaluations of the relative efficiency of management accomplishments, however they may have been planned or executed. Mathematical programming is thereby extended for use as a tool for control and evaluation of past accomplishments as well as a tool to aid in planning future activities. The CCR ratio form introduced by Charnes, Cooper and Rhodes, as part of their Data Envelopment Analysis approach, comprehends both technical and scale inefficiencies via the optimal value of the ratio form, as obtained directly from the data without requiring a priori specification of weights and/or explicit delineation of assumed functional forms of relations between inputs and outputs. A separation into technical and scale efficiencies is accomplished by the methods developed in this paper without altering the latter conditions for use of DEA directly on observational data. Technical inefficiencies are identified with failures to achieve best possible output levels and/or usage of excessive amounts of inputs. Methods for identifying and correcting the magnitudes of these inefficiencies, as supplied in prior work, are illustrated. In the present paper, a new separate variable is introduced which makes it possible to determine whether operations were conducted in regions of increasing, constant or decreasing returns to scale (in multiple input and multiple output situations DMUs that produce more with the least use of inputs are considered efficient (with a score of 100.0%). The linear combination of inputs and outputs from these efficient units forms an efficient or productive frontier, which serves as a performance benchmark for the others. It is worth highlighting that DEA provides pathways for inefficient units to become efficient, through increased production or input reduction. DEA is frequently used in the health sector and has been applied to Brazilian hospitals for different purposes: efficiency analysi^s^,^7^ search for efficiency determinan^t^s^8^ and assessment of public policy performanc^e^.^9^

The impact of the COVID-19 pandemic on the productivity of general THs in Brazil has not been measured yet, nor has it been determined whether this impact occurred uniformly. Following the introduction of vaccination in Brazil, in 2021, and the end of the global health emergency due to COVID-19, in 2023, the epidemiological profile of hospital admissions in general THs gradually returned to pre -pandemic levels, with a predominance of chronic-degenerative disease^s.^ 10 Thus, the productivity of general HEs must be restored in order to recover their role in providing high-complexity care in the national level.

This study aims to analyze the influence of the COVID-19 pandemic on the productivity of general THs in Brazil, by region and legal entity, and to propose parameters of care. 

## METHODS

### Study design and setting

THs have changed their healthcare delivery patterns to cope with the pandemic. An observational and analytical study on the comparative productivity of general THs in Brazil, before (2019) and during (2021) the COVID-19 pandemic, according to region of the country and legal entity, was conducted using non-parametric linear programming modeling, data envelopment analysis (DEA).

### Participants and study size

The study included all THs in Brazil, certified in 2019 and 2021, excluding specialized hospitals and maternity hospitals. The general THs were classified according to the legal entity of their management and grouped based on the Brazilian Institute of Geography and Statistics categorization: direct public administration; corporate entity (public company under private law and private company); and private non-profit entit^y.^ 11 General THs were also classified according to the number of beds: medium-sized hospitals (51 to 150 beds) and large-sized hospitals (over 150 beds).

### Variables

For the mathematical model, variables were selected based on their regular use in similar articl^es^ 12 and their availability in the administrative databases of the Brazilian National Health System (*Sistema Único de Saúde* - SUS).

Input variables included: number of hospital ward beds, number of ICU beds, the specialized services index and hospital mortality rate (HMR). In order to calculate the specialized services index – a measure of the complexity provided – a panel of experts (managers and epidemiologists) was invited to assign a score from 1 to 5 for each procedure included in the SUS table for high-complexity procedures, taking into account the following criteria: complexity (level of professional expertise required), cost (expenditure of physical and financial resources) and procedure duration/hospitalization time (for surgical procedures, the duration of the procedure; for clinical procedures, the average length of stay). Each hospital received a score based on the sum of its qualifications, weighted by complexity. This methodology was described in a previous stud^y.^ 13 HMR is the percentage of deaths (from any cause) regarding the total number of hospital discharges and deaths.

The output variables were: number of hospitalizations adjusted for hospital complexity and the number of surgeries. For adjusting hospitalizations, the number of hospitalizations for each hospital was multiplied by the ratio between the specialized services index and the national average of the same index.

Specific COVID-19 indicators (external to the model) were: COVID-19 incidence rate (number of confirmed cases per 100,000 inhabitants) and COVID-19 mortality rate (number of deaths due to the disease per 100,000 inhabitants).

### Data sources

Data were obtained from the information systems of the Brazilian National Health System Information Technology Department (*Departamento de Informática do Sistema Único de Saúde*), with support from the Microdatasus packag^e.^ 14 These included: National Health Establishment Registry (*Cadastro Nacional de Estabelecimentos de Saúde*), for inputs and qualifications; Hospital Information System (*Sistema de Informações Hospitalares*), for output data; and Mortality Information System (*Sistema de Informação sobre Mortalidade*), for HMR. Specific COVID-19 indicators were obtained from the Coronavirus Panel (https://covid.saude.gov.br) of the Ministry of Health. Data from January to December of 2019 and 2021 were used, accessed in July 2023.

### Mathematical method: DEA-Malmquist

Efficiency scores for general THs and output parameters for recovery planning were calculated by constructing efficient frontiers, based on data from 2019 and 2021, using DEA models.

The classical DEA model with variable returns to scale (VRS) was chosen due to the differences in scale among the DMUs. The distance between the observed DMU and its feasible projection point on the Pareto-efficient frontier (Russel measu^re^ 15 ) was used to calculate the efficiency score of inefficient DMUs and to define parameters of care. It is worth noting that in order to propose these parameters, the efficient frontier incorporated two distinct epidemiological scenarios: before and during the pandemic. The study adopted an output-oriented approach, given that efficiency improvement in Brazilian public health is achieved by increasing output, rather than reducing inputs.

The DEA-Malmquist ind^ex^ 16 assessed the displacement of the efficient frontier between the two different periods – 2019 and 2021 – by calculating the distances between each observed DMU and both frontiers. Index values greater than 1.00 indicate productivity growth; values less than 1.00 indicate a decline. 

The DEA-Malmquist index was decomposed to evaluate two distinct sources of productivity variation: change in technical efficiency (catchup) and change in technological efficiency (frontier-shift). The former indicates a change in the relative efficiency of the same DMU over time. The later represents the overall shift of the frontier, indicating either contraction or expansion, i.e., a decline or improvement in the productivity of the units as a whole.

The DEA-Malmquist and Russel measures were constructed in spreadsheets and programmed using the Solver add-in for Microsoft® Excel.

### Ethical aspects

The study Implementation of operational research models in hospital planning and management, from which this article was derived, was approved by the Research Ethics Committee of the Instituto de Estudos em Saúde Coletiva da Universidade Federal do Rio de Janeiro, CAAE: 41480720.6.0000.5286, o**n** March 9, 2021.

## RESULTS

A total of 213 THs was identified, 64 (30.0%) of which were excluded from the analysis because they were specialized hospitals and maternity hospitals. Among the general THs, 103 (69.1%) hospitals were located in the Southeast and South regions and 131 (87.9%) were large hospitals. Regarding the incidence and specific mortality due to COVID-19, there was a predominance in the South and Midwest regions, followed by the Southeast region (Table 1).

**Table 1:** Number of general teaching hospitals, by size, and incidence of COVID-19 cases and deaths, by region of the country, Brazil, 2021

**Region of the country**	**Hospitals**			**Incidence of COVID-19** **(/100,000 inhabitants)**	**COVID-19 mortality rate** **(/100,000 inhabitants)**
	**Medium size**	**Large size**	**Total**		
Midwest	1	10	11	9,364.4	254.2
North East	4	23	27	5,341.1	126.5
North	1	7	8	5,768.8	160.0
Southeast	11	58	69	6,750.0	232.2
South	1	33	34	9,955.5	251.3
Brazil	18	131	149	6,941.3	201.6

Table 2 presents the inputs and output of Brazil’s general THs in the study period. Of the total number of beds in the country’s general THs (ward and ICU beds combined), the Southeast and South regions accounted for 71.5%, in 2019, and 71.4% in 2021. Between 2019 and 2021, there was a reduction of 604 (-1.4%) ward beds and an increase of 4,614 (51.9%) ICU beds. The supply of hospital admissions for high-complexity procedures, assessed by the specialized services index, increased by 1.5%. On the other hand, there was a decline of 299,547 (-11.6%) in hospitalizations adjusted for complexity and 78,431 (-8.8%) in surgeries performed. The HMR increased by 2.7 percentage points.

**Table 2:** Total inputs and output of teaching hospitals before and after the COVID-19 pandemic, by region and legal entity, Brazil, 2019 and 2021

**Classification of teaching hospitals**		**Inputs**						**Outputs**					
**Region/Legal entity**	**N**	**Ward beds**		**ICU beds**		**Average** **specialized services index**		**Hospitalizations adjusted for complexity**		**Surgeries**		**Hospital mortality**	
		**2019**	**2021**	**2019**	**2021**	**2019**	**2021**	**2019**	**2021**	**2019**	**2021**	**2019**	**2021**
**Midwest**													
Public administration	6	1,835	1,815	301	525	37	38	65,503	58,538	31,149	28,953	5.3	6.8
Corporate entity	4	663	696	127	268	64	63	40,232	37,647	14,484	14,311	3.6	5.2
Private non-profit entities	1	296	296	35	38	56	56	8,449	8,382	5,062	4,431	5.2	7.2
Total	11	2,794	2,807	463	831	48	49	114,184	104,567	50,695	47,695	4.7	6.2
**Northeast**													
Public administration	11	4,027	4,217	818	1,036	44	46	144,182	145,224	71,368	74,598	6.3	7.8
Corporate entity	11	2,239	2,287	364	499	59	64	108,211	97,370	41,827	41,081	4.7	5.7
Private non-profit entities	5	2,199	2,253	229	285	58	58	55,293	48,730	32,785	30,970	7.2	8.2
Total	27	8,465	8,757	1,411	1,820	53	55	307,685	291,324	145,980	146,649	5.8	7.0
**North**													
Public administration	6	1,290	1,267	333	516	35	36	25,139	28,301	17,249	23,851	10.7	12.1
Corporate entity	2	423	376	35	67	36	39	4,456	4,237	4,441	3,123	8.9	10.0
Total	8	1,713	1,643	368	583	35	37	29,594	32,538	21,690	26,974	10.2	11.6
**Southeast**													
Public administration	32	9,593	9,569	2,095	3,174	60	60	636,471	534,099	204,356	179,058	5.5	8.3
Corporate entity	9	2,551	2,454	520	720	79	78	173,573	143,974	54,550	46,559	4.7	7.0
Private non-profit entities	28	8,538	8,342	1,649	2,749	77	78	549,654	540,095	160,343	153,543	9.1	12.7
Total	69	20,682	20,365	4,264	6,643	69	70	1,359,699	1,218,168	419,249	379,160	6.8	9.9
**South**													
Public administration	9	1,860	2011	401	846	59	60	115,911	121,342	63,149	63,573	4.2	7.8
Corporate entity	8	3,526	3,305	987	1,343	88	88	333,206	237,407	74,450	52,099	4.4	6.8
Private non-profit entities	17	5,532	5,080	993	1,435	83	84	326,185	281,573	115,100	95,732	8.0	12.8
Total	34	10,918	10,396	2,381	3,624	78	79	775,303	640,321	252,699	211,404	6.1	10.1
**Brazil**													
Public administration	64	18,605	18,879	3,948	6,097	53	53	987,206	887,503	387,271	370,033	5.9	8.3
Corporate entity	34	9,402	9,118	2,033	2,897	70	72	659,678	520,636	189,752	157,173	4.8	6.5
Private non-profit entities	51	16,565	15,971	2,906	4,507	77	78	939,582	878,779	313,290	284,676	8.4	12.2
Total	149	44,572	43,968	8,887	13,501	65	66	2,586,465	2,286,918	890,313	811,882	6.5	9.2

The reduction in ward beds was greater in hospitals in the South (-4.8%) and North (-4.1%) regions, while an increase was observed in the Northeast region (3.4%). The number of ICU beds increased across all regions, ranging from 29.0% in the Northeast region to 79.5% in the Midwest region. As for output, the South and Southeast regions were the most affected by the pandemic, with a reduction in hospitalizations (-17.4% and -10.4%, respectively) and surgeries (-16.3% and -9.6% %, respectively). Only the North region showed an increase in output: 9.9% in hospitalizations and 24.4% in surgeries. It is also worth highlighting that the North region had a high HMR, even before the pandemic (10.2% in 2019), and the impact of the pandemic on HMR in the South region (from 6.1% to 10.1%).

Regarding legal entity, public hospitals under direct administration, corporate hospitals and private non-profit hospitals accounted for 43.0%, 22.8% and 34.2% of the country’s general THs, respectively. There was a reduction in ward beds in corporate hospitals (-3.0%) and in private non-profit hospitals (-3.6%), and an increase in public hospitals under direct administration (1.5%). General THs of all legal entity types increased the number of ICU beds: 54.4% in direct administration public hospitals; 42.5% in corporate hospitals; and 55.1% in private, non-profit hospitals. Regarding output, all general THs showed a reduction in hospitalizations and surgeries, respectively: -10.1% and - 4.5% in direct administration public institutions; -21.1% and -17.2% in corporate hospitals; and -9.1% and -6.5% in private non-profit hospitals. The HMR increased by 2.4%, 1.7%, 3.8% in public, corporate and private non-profit hospitals, respectively, with the highest rates found among private non-profit hospitals (8.4% in 2019 and 12.2% in 2021).

Table 3 shows the efficiency results of general THs, in 2019 and 2021, as well as the frontier shift over the period (Malmquist index). It could be seen an increase in the average relative efficiency scores of general THs in the Midwest, Northeast and North regions. In 2019, general THs in the Northeast region were the most efficient (58.1%); in 2021, The most efficient general THs were in the Northeast and Midwest regions (59.4%). General THs in the North region remained the least efficient in both years analyzed (36.7%, in 2019, and 42.8%, in 2021), despite the increased output during this period. During the pandemic, direct administration general THs were more efficient in the North and South regions; corporate general THs, in the Midwest and Southeast regions; and private non-profit general THs, in the Northeast region. Despite the observed increase in general TH efficiency (catch-up 1.01), the productivity frontier contracted (Malmquist 0.77; frontier-shift 0.76) in all regions (Malmquist ranging from 0.67 to 0.92; frontier-shift from 0.66 to 0.81) and across all legal entity types (Malmquist from 0.62 to 0.88; frontier-shift from 0.58 to 0.88). Only direct administration public hospitals in the Midwest region showed a Malmquist index greater than 1.00 (equal to 1.01), however, with *a* frontier-shift of 0.85.

**Table 3:** Average efficiency of teaching hospitals and Malmquist index, by region of the country and legal entity, Brazil, 2019 and 2021

Classification of teaching hospitals	**Efficiency 2019 (%)**	**Efficiency 2021 (%)**	**Malmquist Index**	**Technical efficiency** **(Catch-up)**	**Technological efficiency (Frontier-shift)**
**Region/Legal entity**
**Midwest**					
Public administration	47.6	56.7	1.01	1.19	0.85
Corporate entity	54.3	67.8	0.81	1.25	0.65
Private non-profit entity	39.5	41.7	0.85	1.06	0.81
Average	49.3	59.4	0.92	1.20	0.76
**Northeast**					
Public administration	49.7	56.8	0.90	1.14	0.79
Corporate entity	65.6	60.0	0.53	0.91	0.58
Private non-profit entity	59.9	63.5	0.71	1.06	0.68
Average	58.1	59.4	0.67	1.02	0.66
**North**					
Public administration	36.3	50.1	0.96	1.38	070
Corporate entity	37.6	20.8	0.35	0.55	0.63
Average	36.7	42.8	0.78	1.17	0.67
**Southeast**					
Public administration	57.7	59.0	0.90	1.02	0.88
Corporate entity	64.2	59.8	0.69	0.93	0.74
Private non-profit entity	55.9	55.6	0.74	1.00	0.74
Average	57.8	57.7	0.81	1.00	0.81
**South**					
Public administration	69.1	66.4	0.71	0.96	0.74
Corporate entity	57.2	56.3	0.68	0.99	0.69
Private non-profit entity	48.2	46.0	0.71	0.96	0.74
Average	55.8	53.8	0.70	0.96	0.73
**Brazil**					
Public administration	55.0	58.6	0.88	1.07	0.82
Business entity	60.3	57.7	0.62	0.96	0.64
Private non-profit entity	53.4	52.9	0.73	0.99	0.74
Average	55.6	56.5	0.77	1.01	0.76

32 efficient units were identified, as benchmarks for inefficient general THs (Supplementary Material). A total of 15 general THs was efficient in both years analyzed. Among the benchmarks, 24 (75.0%) were large hospitals, 16 (50.0%) were located in the Southeast region and 11 (34.4%) were private, non-profit hospitals.

Table 4 presents the expected output projection for all inefficient general THs to reach the best practice frontier as of 2021. In a recovery scenario, given the current availability of inputs, these hospitals should increase their output by 2,205,856 (96.5%) hospitalizations and 872,264 (107.4%) surgeries. Simultaneously, with the end of the global health emergency due to COVID-19, a decrease in the HMR from 9.2% to 4.0% is expected, particularly in the North region (from 11.6% to 3.5%) and among private non-profit hospitals (from 12.2% to 4.0%).

**Table 4:** Expected projection for service output and the estimated mortality rate of teaching hospitals after the COVID-19 pandemic, by region and legal entity, Brazil, 2021

**Classification of teaching hospitals**	**Hospitalizations**	**Projection of hospitalizations**	**Difference (%)**	**Surgeries**	**Projection of surgeries**	**Difference (%)**	**Hospital mortality**	**Hospital mortality projection**
**Region/Legal entity**
**Midwest**								
Public administration	58,538	145,076	147.8	28,953	65,135	125.0	6.8	2.6
Corporate entity	37,648	85,191	126.3	14,311	31,162	117.7	5.2	4.0
Private non-profit entity	8,382	21,413	155.5	4,431	11,319	155.5	7.2	3.4
Total	104,568	251,680	140.7	47,695	107,617	125.6	6.2	3.2
**Northeast**								
Public administration	145,223	306,072	110.8	74,598	142,563	91.1	7.8	4.5
Corporate entity	97,370	200,557	106.0	41,081	88,781	116.1	5.7	4.1
Private non-profit entity	48,730	107,156	119.9	30,970	59,748	92.9	8.2	3.8
Total	291,323	613,786	110.7	146,649	291,092	98.5	7.0	4.2
**North**								
Public administration	28,302	81,905	189.4	23,851	48,840	104.8	12.1	3.4
Corporate entity	4,237	23,100	445.2	3,123	17,118	448.1	10.0	3.8
Total	32,539	105,005	222.7	26,974	65,958	144.5	11.6	3.5
**Southeast**								
Public administration	534,099	1,001,530	87.5	179,058	359,894	101.0	8.3	3.6
Corporate entity	143,974	278,643	93.5	46,559	99,817	114.4	7.0	4.6
Private non-profit entity	540,095	966,618	79.0	153,543	313,619	104.3	12.7	4.5
Total	1,218,168	2,246,790	84.4	379,160	773,330	104.0	9.9	4.1
**South**								
Public administration	121,340	222,276	83.2	63,573	105,074	65.3	7.8	4.4
Corporate entity	237,407	463,996	95.4	52,099	124,809	139.6	6.8	4.2
Private non-profit entity	281,572	589,240	109.3	95,732	216,266	125.9	12.8	3.5
Total	640,319	1,275,512	99.2	211,404	446,149	111.0	10.1	3.9
**Brazil**								
Public administration	887,502	1,756,859	98.0	370,033	721,506	95.0	8.3	3.7
Corporate entity	520,636	1,051,486	102.0	157,173	361,687	130.1	6.5	4.2
Non-profit entity	878,779	1,684,427	91.7	284,676	600,953	111.1	12.2	4.0
Total	2,286,917	4,492,773	96.5	811,882	1,684,146	107.4	9.2	4.0

## DISCUSSION

In this study, a decline in the productivity of general THs in Brazil across all regions and for all legal entity types was observed, in the period from 2019 to 2021. It is worth highlighting that this decline in productivity was due to the social commitment of general THs rather than a failure in public policy. In other words, the increase in the supply of inputs (ICU beds) and the drop in output (hospitalizations and surgeries) occurred in response to the strategic actions required to combat the pandemic. Similarly, the observed increase in HMR during the pandemic, was an indicator of the frequency of COVID-19 hospitalizations in the units under analysis, not a reflection of the quality of care provided.

The reduction in production, with less attention to other diseases, was a global phenomeno^n.^ 17 Taking into consideration the central role of THs, responsible for 35.3% of high-complexity production in the countr^y^,^2^ the decline in production had a significant impact on the performance of more complex procedures. In 2021, the country’s general THs reduced heart surgeries by 14.8%, cancer surgeries by 8.4%, radiotherapy by 96.8% and transplants by 18.6^%^.^2^ Estimates indicated a backlog of 60,000 cardiovascular surgeries due to the pandemic, further increasing the surgical waiting lis^t.^ 18 The reduction in output, at all levels of care, had a widespread effect on the health of the Brazilian population; for example, mortality from cardiovascular diseases increased by 6.9% in the same perio^d^.^6^ In addition, teaching and research activities were also compromised, with the exception of academic work aimed at COVID-19.

In order to restore productivity, the number of hospitalizations and surgeries needs to nearly double nationwide, and managers of each health unit (as well as those from municipalities and states) can plan how much they need to increase output. It is worth highlighting that the activation of 4,814 (51.9%) ICU beds (above the national average of 46.7%) supports the high-complexity production role of general THs as they resume these procedures. In other words, additional inputs to support high-complexity care promote increased output, as long as the units remain at the efficient frontier.

As a limitation of the study, it is worth mentioning the absence of qualitative models to structure the problem before the mathematical modeling. Studi^es19,20,^ 21 suggest the use of associated methodologies (multimethodology) to contexts and preference assessment before choosing mathematical models. Regarding the model variables, there was a lack of accurate information on the hiring of human resources, which was important for productivity during the pandemic, and could have been included in the model. Data on teaching and research activities would also have enriched the analysis, since the volume of research, high resident-to-bed ratio (teaching intensity) and low resident-to-physician ratio (teaching dedication) are associated with increased efficienc^y.^ 22 Another limitation lies in the heterogeneity of information among Brazilian regions. A study on excess mortality during the pandem^ic^ 23 suggests greater diagnostic challenges and underreporting of deaths due to COVID-19 in Northeastern capitals compared to Southeastern capitals. Research using network DEA to study the capacity and structures for addressing COVID-19 showed that the North and Northeast regions were more vulnerable during the pandemic due to a lack of structure (ICU beds) and lower capacity to reallocate resources (doctors and ventilators) to meet the excess demand from people with COVID-1^9.^ 24

As a recommendation for further research, the DEA-Malmquist model could be applied in the years following this study, given the introduction of vaccines nationwide (starting in 2021), and the emergence of successive waves and new strains of COVID-19. To improve the study of reference units, qualitative and quantitative research can identify patterns of successful strategies employed during the pandemic. The use of this tool, along with a better characterization of the output of these hospitals, will allow for the estimation and monitoring of the gradual recovery of service delivery in line with the evolving needs and demands of Brazilian society.

## Apêndice

**Supplementary Table 1:** Efficient General Teaching Hospitals by region, state, legal entity, and size, Brazil, 2019 and 2021

Hospital name	Region	State	Legal entity	Size	Reference (years)
Fundação Hospital Adriano Jorge	North	AM	Public Administration	Large	2019 and 2021
Hospital Anchieta	Southeast	SP	Public Administration	Medium	2019 and 2021
Hospital da Baleia	Southeast	MG	Private Non-Profit Entity	Medium	2019 and 2021
Hospital da Restauração Governador Paulo Guerra	Northeast	PE	Public Administration	Large	2019 and 2021
Hospital das Clínicas da Faculdade de Medicina de Ribeirão Preto	Southeast	SP	Public Administration	Large	2019 and 2021
Hospital das Clínicas da Faculdade de Medicina de São Paulo	Southeast	SP	Public Administration	Large	2019 and 2021
Hospital das Clínicas da Universidade Federal de Pernambuco (EBSERH^a^)	Northeast	PE	Corporate Entity	Large	2021
Hospital de Base de São José do Rio Preto	Southeast	SP	Private Non-Profit Entity	Large	2021
Hospital de Caridade São Vicente de Paulo	Southeast	SP	Private Non-Profit Entity	Large	2019 and 2021
Hospital do Trabalhador	South	PR	Public Administration	Large	2019 and 2021
Hospital Estadual de Sumaré Dr. Leandro Franceschini	Southeast	SP	Public Administration	Large	2021
Hospital Geral do Grajau	Southeast	SP	Public Administration	Large	2019
Hospital Getúlio Vargas	Northeast	PE	Public Administration	Large	2019 and 2021
Hospital Municipal da Piedade	Southeast	RJ	Public Administration	Medium	2019 and 2021
Hospital Municipal Universitário de São Bernardo do Campo	Southeast	SP	Public Administration	Large	2019 and 2021
Hospital Nossa Senhora do Rócio	South	PR	Corporate Entity	Large	2019
Hospital Regional do Paranoá	Central-West	DF	Public Administration	Large	2021
Hospital Santa Lucinda da Pontifícia Universidade Católica de São Paulo	Southeast	SP	Private Non-Profit Entity	Medium	2021
Hospital Universitário Cajuru da Pontifícia Universidade Católica do Paraná	South	PR	Private Non-Profit Entity	Large	2019 and 2021
Hospital Universitário Clemente de Faria da Universidade Estadual de Montes Claros	Southeast	MG	Public Administration	Large	2021
Hospital Universitário da Universidade Federal do Maranhão (EBSERH^a^)	Northeast	MA	Corporate Entity	Large	2021
Hospital Universitário da Universidade Federal de Sergipe (EBSERH^a^)	Northeast	SE	Corporate Entity	Medium	2019
Hospital Universitário da Universidade Federal de São Carlos (EBSERH^a^)	Southeast	SP	Corporate Entity	Medium	2019 and 2021
Hospital Universitário de Lagarto da Universidade Federal de Sergipe (EBSERH^a^)	Northeast	SE	Corporate Entity	Medium	2019
Hospital Universitário Evangélico Mackenzie	South	PR	Private Non-Profit Entity	Large	2021
Hospital Universitário Júlio Muller da Universidade Federal do Mato Grosso (EBSERH^a^)	Central-West	MT	Corporate Entity	Medium	2021
Hospital Universitário Walter Cantídio da Universidade Federal do Ceará (EBSERH^a^)	Northeast	CE	Corporate Entity	Large	2019 and 2021
Irmandade Nossa Senhora das Mercês de Montes Claros	Southeast	MG	Private Non-Profit Entity	Large	2019
Santa Casa de Misericórdia de Fortaleza	Northeast	CE	Private Non-Profit Entity	Large	2019 and 2021
Santa Casa de Misericórdia de Limeira	Southeast	SP	Private Non-Profit Entity	Large	2019
Santa Casa de Misericórdia do Pará	North	PA	Private Non-Profit Entity	Large	2021
Santa Casa de Misericórdia de Belo Horizonte	Southeast	MG	Private Non-Profit Entity	Large	2021

a) Empresa Brasileira de Serviços Hospitalares.
