# Supplementary material for: Assessment of the impact of the COVID-19 pandemic on the productivity of teaching hospitals in Brazil
Source: Epidemiol Serv Saude. 2024 Dec 6;33:e20231176. doi: 10.1590/S2237-96222024v33e20231176.en (PMC11654046; doi:10.1590/S2237-96222024v33e20231176.en)
Supplement: Translation

# Translation: Avaliação do impacto da pandemia de covid-19 na produtividade dos hospitais de ensino no Brasil

**DOI:** 10.1590/S2237-96222024v33e20231176.pt

**Authors:** Maria Stella de Castro Lobo, Marcos Pereira Estellita Lins, Henrique de Castro Rodrigues, Gabriel Martins Soares

**Affiliations:** 1 Universidade Federal do Rio de Janeiro, Instituto de Estudos em Saúde Coletiva, Rio de Janeiro, RJ, Brasil; 2 Universidade Federal do Estado do Rio de Janeiro, Escola de Engenharia de Produção, Rio de Janeiro, RJ, Brasil; 3 Universidade Federal do Rio de Janeiro, Instituto Alberto Luiz Coimbra de Pós-Graduação e Pesquisa de Engenharia, Rio de Janeiro, RJ, Brasil; 4 Hospital Universitário Clementino Fraga Filho, Serviço de Epidemiologia e Avaliação e Laboratório de Informação para Gestão Hospitalar, Rio de Janeiro, RJ, Brasil

**Keywords:** Hospitais de Ensino, Pesquisa Operacional, Benchmarking, Eficiência Organizacional, Covid-19

## Abstract

**Objetivos:**

Analisar a influência da pandemia de covid-19 na produtividade dos hospitais gerais de ensino do Brasil, por região e natureza jurídica, e propor parâmetros assistenciais.

**Métodos:**

Estudo observacional por modelagem matemática com análise envoltória de dados e índice de Malmquist, utilizando dados de recursos e produção assistencial antes (2019) e durante (2021) a pandemia.

**Resultados:**

Foram analisados 149 hospitais gerais de ensino, 32 dos quais foram considerados eficientes. Houve queda da produtividade em todas regiões e naturezas jurídicas. Para que todos os hospitais ineficientes atinjam a fronteira de eficiência gerada pela modelagem, há necessidade de aumento da produção em 2.205.856 (96,5%) internações e 872.264 (107,4%) cirurgias.

**Conclusão:**

A queda na produtividade hospitalar decorreu do compromisso social dos hospitais durante a pandemia, com modifica*ção* do padrão de assistência. O modelo matemático utilizado permite gerar parâmetros para recuperação assistencial eficiente depois de finalizada emergência sanitária, podendo ser aplicado para planejamento hospitalar.

## Contribuições do estudo

**Principais resultados:**

A pandemia de covid-19 reduziu a produtividade dos hospitais gerais de ensino do Brasil em todas as regiões e em todas as naturezas jurídicas. Para a recuperação, os hospitais devem elevar, em média, 96,5% de internações e 107,4% de cirurgias.

**Implicações para os serviços:**

O estudo apresenta metodologia que pode ser adaptada e replicada na gestão de serviços de saúde do país, ao definir um escore de eficiência e calcular os parâmetros, num cenário de recuperação assistencial após o fim da emergência em saúde pública.

**Perspectivas:**

Análise qualitativa adicional e aplicação de DEA-Malmquist, nos anos subsequentes, validarão o planejamento dinâmico, que considera múltiplas oscilações e a influência de novos fatores e contextos que alteram a produtividade (caso da pandemia).

## INTRODUÇÃO

O enfrentamento da pandemia de covid-19 demandou esforço conjunto da sociedade e da saúde pública nacional. Recursos federais, estaduais e municipais da saúde foram somados para a contratação de profissionais de saúde e aquisição de equipamentos médicos, como oxigênio, sedativos e equipamentos de proteção individua^l^.^1^ Considerando-se a alta transmissibilidade e potencial de gravidade nos dois primeiros anos da pandemia, houve aumento de 47,0% dos leitos de centros de terapia intensiva (CTIs) e de 4,7% dos demais leitos, além da instalação de hospitais de campanha e reconfiguração de unidades para atendimento exclusivo a pacientes acometidos por covid-1^9.1^,^2^

Hospitais de ensino (HEs) participaram desse processo com diferentes estratégias, como suspensão de consultas e cirurgias eletivas, ampliação de leitos de terapia intensiva, reforço das rotinas de biossegurança, contratação de novos profissionais, capacitação das equipes de saúde, suspensão das atividades de ensino e desenvolvimento de pesquisas sobre o tem^a^.^3^

Note-se que a adoção de medidas de resposta à pandemia representou renúncias no tratamento de outras doença^s^,^4^ com consequentes alterações no perfil das admissões e na eficiência produtiva. Produtividade é a relação entre o volume de produtos fornecidos e de recursos utilizados por uma mesma unidade produtiva. Eficiência técnica é medida quando comparada a produtividade de unidades semelhantes, para se avaliar o potencial máximo da produção em relação aos recursos disponívei^s^.^5^ Existem diferentes métodos para a análise de produtividade, como o método dos mínimos quadrados, a produtividade total dos fatores, a análise de fronteira estocástica e a análise envoltória de dados (*data envelopment analysis –* DEA^)^.^5^

A DEA é uma técnica de programação linear que permite medir o desempenho de unidades produtivas, denominadas unidades tomadoras de decisão (*decision making units – DMU*), que consomem múltiplos recursos – como leitos, equipamentos e recursos humanos – para gerarem variados produtos, a exemplo de internações, cirurgias e consulta^s^.^6^ As DMUs que produzem mais com o menor uso de recursos são consideradas eficientes (escore igual a 100,0%). A combinação linear de recursos e produtos destas unidades eficientes desenha uma fronteira de eficiência ou produtividade, a qual se constitui em referência de desempenho para as demais. A DEA fornece os caminhos para que as unidades ineficientes se tornem eficientes, por meio de aumento da produção ou redução de recursos. Frequentemente usada na área da saúde, a DEA já foi utilizada em hospitais do Brasil com objetivos distintos: análise de eficiênci^a^,^7^ busca de fatores determinantes de eficiênc^i^a^8^ e avaliação do desempenho de políticas pública^s^.^9^

Ainda não foram mensurados os impactos provocados pela pandemia de covid-19 na produtividade dos HEs gerais no Brasil, tampouco se esse impacto se deu de forma homogênea. Após a introdução da vacinação no Brasil, em 2021, e o fim da emergência global em saúde por covid-19, em 2023, o perfil epidemiológico das admissões nos HEs gerais retornou paulatinamente aos níveis pré-pandêmicos, com predominância das doenças crônico-degenerativa^s.^ 10 Assim, a produtividade dos HEs gerais deve ser retomada para a recuperação de seu papel de atenção de alta complexidade no cenário nacional. 

Este estudo objetiva analisar a influência da pandemia de covid-19 na produtividade dos HEs gerais do Brasil, por região e natureza jurídica, e propor parâmetros assistenciais. 

## MÉTODOS

### Delineamento e contexto

HEs alteraram seus padrões de atenção em saúde para lidar com a pandemia. Estudo observacional e analítico sobre a produtividade comparada dos HEs gerais do Brasil, antes (2019) e durante (2021) a pandemia de covid-19, de acordo com região do país e natureza jurídica, foi desenvolvido por meio de modelagem de programação linear não-paramétrica, análise envoltória de dados (DEA). 

### Participantes e tamanho do estudo

Foi considerada a totalidade dos HEs do Brasil, assim habilitados nos anos de 2019 e 2021, sendo excluídos os hospitais especializados e maternidades. Os HEs gerais foram classificados quanto à natureza jurídica da gestão e agrupados com base na categorização do Instituto Brasileiro de Geografia e Estatística: administração pública direta; entidades empresariais (empresa pública de direito privado e empresa privada); e entidades privadas sem fins lucrativo^s.^ 11 Os HEs gerais também foram classificados quanto ao número de leitos: médio porte (de 51 a 150 leitos) e grande porte (acima de 150 leitos).

### Variáveis

Para o modelo matemático, variáveis foram selecionadas pela regularidade com que são utilizadas em artigos similar^es^ 12 e disponibilidade nas bases de dados administrativas do Sistema Único de Saúde (SUS).

As variáveis de recursos foram: número de leitos de enfermaria, número de leitos de CTI, índice de serviços especializados e taxa de mortalidade hospitalar (TMH). Para cálculo do índice de serviços especializados – uma medida da complexidade ofertada –, um painel de expertos (gestores e epidemiologistas) foi convidado a dar uma nota de 1 a 5 para cada procedimento constante na tabela de habilitações do SUS específicas para alta complexidade, levando em consideração os seguintes critérios: complexidade (nível de *expertise* profissional necessária para sua realização), custo (dispêndio de recursos físicos e financeiros) e tempos de realização dos procedimentos/internação necessários (no caso de procedimentos cirúrgicos, duração do procedimento; no caso de procedimentos clínicos, o tempo médio de internação). Cada hospital recebeu a pontuação de acordo com o somatório de suas habilitações, ponderadas pela complexidade. Essa metodologia foi descrita em trabalho anterio^r.^ 13 A TMH é o percentual de óbitos (por qualquer causa) em relação ao total de saídas hospitalares (óbitos e altas).

As variáveis de produção foram: número de internações ajustadas por complexidade do hospital e número de cirurgias. Para o ajuste das internações, o número de internações de cada hospital foi multiplicado pela razão entre o índice de serviços especializados e a média nacional do mesmo índice.

Indicadores específicos de covid-19 (externos ao modelo) foram: taxa de incidência por covid-19 (número de casos confirmados por 100 mil habitantes) e taxa de mortalidade por covid-19 (número de óbitos pela doença por 100 mil habitantes).

### Fontes de dados

Os dados foram obtidos nos sistemas de informação do Departamento de Informática do Sistema Único de Saúde, com apoio do pacote Microdatasu^s.^ 14 Entre eles: Cadastro Nacional de Estabelecimentos de Saúde, para recursos e habilitações; Sistema de Informações Hospitalares, para produção; e Sistema de Informação sobre Mortalidade, para TMH. Indicadores específicos de covid-19 foram obtidos no Painel Coronavírus (https://covid.saude.gov.br) do Ministério da Saúde. Utilizaram-se os meses de janeiro a dezembro de 2019 e de 2021. Os dados foram acessados em julho de 2023.

### Método matemático: DEA-Malmquist

Escores de eficiência dos HEs gerais e parâmetros de produção para planejamento da recuperação foram calculados a partir da construção de fronteiras de eficiência, com base nos dados de 2019 e 2021, utilizando-se modelos de DEA.

Optou-se pelo modelo clássico DEA, com retornos variáveis de escala (VRS), pelas diferenças de escala entre as DMUs. A distância entre a DMU observada e seu ponto de projeção viável na fronteira Pareto-eficiente (medida de Russ^el^ 15) foi utilizada para cálculo do escore de eficiência das DMUs ineficientes e para definição de parâmetros assistenciais. Observe-se que, para proposição destes parâmetros, a fronteira de eficiência incorporou dois cenários epidemiológicos distintos: antes e durante a pandemia. O estudo adotou orientação a produto (*output*), considerando-se que a melhoria de eficiência na saúde pública brasileira se dá pelo aumento de produção, e não pela redução de recursos. 

O índice de DEA-Malmqui^st^ 16 avaliou o deslocamento da fronteira de eficiência entre os dois momentos distintos – 2019 e 2021 – por meio do cálculo das distâncias entre cada DMU observada e ambas as fronteiras . Valores do índice maiores que 1,00 indicam crescimento da produtividade; valores menores que 1,00 indicam declínio. 

O índice de DEA-Malmquist foi decomposto para avaliar duas fontes distintas de variação da produtividade: mudança na eficiência técnica (*catch-up*) e mudança na eficiência tecnológica (*frontier-shift*). A primeira indica mudança da eficiência relativa de uma mesma DMU ao longo do tempo. A segunda representa o deslocamento da fronteira como um todo, numa dinâmica de contração ou de expansão, isto é, de piora ou melhoria de produtividade das unidades vistas em conjunto. 

Os modelos DEA-Malmquist e Russel foram construídos em planilhas e programados por meio do suplemento Solver do aplicativo Microsoft® Excel. 

### Aspectos éticos

O estudo *Implementação de modelos de pesquisa operacional no planejamento e gestão hospitalar*, de que se originou este artigo, foi aprovado pelo Comitê de Ética em Pesquisa do Instituto de Estudos em Saúde Coletiva da Universidade Federal do Rio de Janeiro, CAAE: 41480720.6.0000.5286, e**m** 9 de março de 2021. 

## RESULTADOS

Foram identificados 213 HEs, sendo excluídos da análise 64 (30,0%), por se tratar de hospitais especializados e maternidades. Entre os HEs gerais, 103 (69,1%) hospitais estavam localizados nas regiões Sudeste e Sul e 131 (87,9%) eram de grande porte. No tocante às incidências de casos e mortalidade específica por covid-19, houve predomínio nas regiões Sul e Centro-Oeste, seguidas pela região Sudeste (Tabela 1).

**Tabela 1:** Número de hospitais gerais de ensino, por porte, e incidência de casos e mortes por covid-19, por região do país, Brasil, 2021

**Região do país**	**Hospitais**			**Incidência de covid-19** **(/100 mil habitantes)**	**Taxa de mortalidade por covid-19** **(/100 mil habitantes)**
	**Médio porte**	**Grande porte**	**Total**		
Centro-Oeste	1	10	11	9.364,4	254,2
Nordeste	4	23	27	5.341,1	126,5
Norte	1	7	8	5.768,8	160,0
Sudeste	11	58	69	6.750,0	232,2
Sul	1	33	34	9.955,5	251,3
Brasil	18	131	149	6.941,3	201,6

A Tabela 2 apresenta os recursos e a produção dos HEs gerais do Brasil no período. Do total de leitos dos HEs gerais do país (leitos de enfermaria e de CTI somados), as regiões Sudeste e Sul respondiam por 71,5%, em 2019, e 71,4% em 2021. Entre 2019 e 2021, houve redução de 604 (-1,4%) leitos de enfermaria e aumento de 4.614 (51,9%) leitos de CTI. A oferta de internações para procedimentos de alta complexidade, avaliada pelo índice de serviços especializados, teve incremento de 1,5%. Por outro lado, houve queda de 299.547 (-11,6%) internações ajustadas por complexidade e de 78.431 (-8,8%) cirurgias realizadas. Houve aumento da TMH em 2,7 pontos percentuais.

**Tabela 2:** Total de recursos e produção dos hospitais de ensino antes e após a pandemia de covid-19, por região e natureza jurídica, Brasil, 2019-2021

**Classificação dos hospitais de ensino**		**Recursos**						**Produtos**					
**Região/Natureza jurídica**	**N**	**Leitos de enfermaria**		**Leitos de** **CTI**		Índice médio de serviços especializados		**Internações ajustadas por complexidade**		**Cirurgias**		**Mortalidade hospitalar**	
		**2019**	**2021**	**2019**	**2021**	**2019**	**2021**	**2019**	**2021**	**2019**	**2021**	**2019**	**2021**
**Centro-Oeste**													
Administração pública	6	1.835	1.815	301	525	37	38	65.503	58.538	31.149	28.953	5,3	6,8
Entidades empresariais	4	663	696	127	268	64	63	40.232	37.647	14.484	14.311	3,6	5,2
Entidades privadas sem fins lucrativos	1	296	296	35	38	56	56	8.449	8.382	5.062	4.431	5,2	7,2
Total	11	2.794	2.807	463	831	48	49	114.184	104.567	50.695	47.695	4,7	6,2
**Nordeste**													
Administração pública	11	4.027	4.217	818	1.036	44	46	144.182	145.224	71.368	74.598	6,3	7,8
Entidades empresariais	11	2.239	2.287	364	499	59	64	108.211	97.370	41.827	41.081	4,7	5,7
Entidades privadas sem fins lucrativos	5	2.199	2.253	229	285	58	58	55.293	48.730	32.785	30.970	7,2	8,2
Total	27	8.465	8.757	1.411	1.820	53	55	307.685	291.324	145.980	146.649	5,8	7,0
**Norte**													
Administração pública	6	1.290	1.267	333	516	35	36	25.139	28.301	17.249	23.851	10,7	12,1
Entidades empresariais	2	423	376	35	67	36	39	4.456	4.237	4.441	3.123	8,9	10,0
Total	8	1.713	1.643	368	583	35	37	29.594	32.538	21.690	26.974	10,2	11,6
**Sudeste**													
Administração pública	32	9.593	9.569	2.095	3.174	60	60	636.471	534.099	204.356	179.058	5,5	8,3
Entidades empresariais	9	2.551	2.454	520	720	79	78	173.573	143.974	54.550	46.559	4,7	7,0
Entidades privadas sem fins lucrativos	28	8.538	8.342	1.649	2.749	77	78	549.654	540.095	160.343	153.543	9,1	12,7
Total	69	20.682	20.365	4.264	6.643	69	70	1.359.699	1.218.168	419.249	379.160	6,8	9,9
**Sul**													
Administração pública	9	1.860	2.011	401	846	59	60	115.911	121.342	63.149	63.573	4,2	7,8
Entidades empresariais	8	3.526	3.305	987	1.343	88	88	333.206	237.407	74.450	52.099	4,4	6,8
Entidades privadas sem fins lucrativos	17	5.532	5.080	993	1.435	83	84	326.185	281.573	115.100	95.732	8,0	12,8
Total	34	10.918	10.396	2.381	3.624	78	79	775.303	640.321	252.699	211.404	6,1	10,1
**Brasil**													
Administração pública	64	18.605	18.879	3.948	6.097	53	53	987.206	887.503	387.271	370.033	5,9	8,3
Entidades empresariais	34	9.402	9.118	2.033	2.897	70	72	659.678	520.636	189.752	157.173	4,8	6,5
Entidades privadas sem fins lucrativos	51	16.565	15.971	2.906	4.507	77	78	939.582	878.779	313.290	284.676	8,4	12,2
Total	149	44.572	43.968	8.887	13.501	65	66	2.586.465	2.286.918	890.313	811.882	6,5	9,2

A redução de leitos de enfermaria foi maior nos hospitais das regiões Sul (-4,8%) e Norte (-4,1%), tendo havido aumento na região Nordeste (3,4%). Os leitos de CTI aumentaram em todas as regiões, variando de 29,0% no Nordeste a 79,5% na região Centro-Oeste. Na produção, as regiões Sul e Sudeste foram as mais afetadas pela pandemia, com redução na produção de internações (-17,4% e -10,4%, respectivamente) e de cirurgias (-16,3% e -9,6%, respectivamente). Somente houve aumento da produção na região Norte: 9,9% em internações e 24,4% em cirurgias. Vale ainda destacar a alta TMH, na região Norte, mesmo antes da pandemia (10,2% em 2019), e o impacto da pandemia na TMH na região Sul (de 6,1% para 10,1%).

Quanto à natureza jurídica, hospitais públicos da administração direta, hospitais empresariais e hospitais privados sem fins lucrativos correspondiam respectivamente a 43,0%, 22,8% e 34,2% dos HEs gerais do país. Houve redução de leitos de enfermaria nos hospitais empresariais (-3,0%) e nos hospitais privados sem fins lucrativos (-3,6%), e aumento nos hospitais públicos da administração direta (1,5%). Os HEs gerais de todas as naturezas jurídicas aumentaram o número de leitos de CTI: 54,4% nos hospitais públicos de administração direta; 42,5% nos hospitais empresariais; e 55,1% nos hospitais privados sem fins lucrativos. Na produção, todos os HEs gerais tiveram redução nas internações e cirurgias, respectivamente: -10,1% e -4,5% nos públicos de administração direta; -21,1% e -17,2% nos empresariais; e -9,1% e -6,5% nos privados sem fins lucrativos. Houve aumento da TMH de 2,4%, 1,7%, 3,8% nos hospitais de administração pública, nos empresariais e nos privados sem fins lucrativos, respectivamente, sendo as maiores taxas encontradas entre os hospitais privados sem fins lucrativos (8,4%, em 2019, e 12,2%, em 2021).

A Tabela 3 mostra os resultados das eficiências dos HEs gerais, em 2019 e 2021, assim como o deslocamento da fronteira no período (índice Malmquist). Houve aumento nos escores médios de eficiência relativa dos HEs gerais das regiões Centro-Oeste, Nordeste e Norte. Em 2019, os HEs gerais do Nordeste eram os mais eficientes (58,1%); em 2021, foram os HEs gerais das regiões Nordeste e Centro-Oeste (59,4%). Os HEs gerais da região Norte mantiveram-se menos eficientes em ambos os anos analisados (36,7%, em 2019, e 42,8%, em 2021), apesar do aumento na produção no período. Durante a pandemia, HEs gerais de administração direta foram mais eficientes nas regiões Norte e Sul; HEs gerais empresariais, nas regiões Centro-Oeste e Sudeste; e HEs gerais privados sem fins lucrativos, na região Nordeste. Apesar do aumento observado nas eficiências dos HEs gerais (*cath-up* 1,01), houve contração da fronteira de produtividade (Malmquist 0,77; *frontier-shift* 0,76) em todas as regiões (Malmquist de 0,67 a 0,92; *frontier-shift* de 0,66 a 0,81) e em todas as naturezas jurídicas (Malmquist de 0,62 a 0,88; *frontier-shift* de 0,58 a 0,88). Somente os hospitais públicos de administração direta da região Centro-Oeste apresentaram valor de índice de Malmquist superior a 1,00 (igual a 1,01), porém, com *frontier-shift* de 0,85.

**Tabela 3:** Eficiência média dos hospitais de ensino e índice Malmquist, por região do país e natureza jurídica, Brasil, 2019 e 2021

Classificação dos hospitais de ensino	**Eficiência 2019 (%)**	**Eficiência 2021 (%)**	**Índice de Malmquist**	**Eficiência técnica (*Catch-up*)**	**Eficiência tecnológica (*Frontier-shift*)**
**Região/Natureza jurídica**
**Centro-Oeste**					
Administração pública	47,6	56,7	1,01	1,19	0,85
Entidades empresariais	54,3	67,8	0,81	1,25	0,65
Entidades privadas sem fins lucrativos	39,5	41,7	0,85	1,06	0,81
Média	49,3	59,4	0,92	1,20	0,76
**Nordeste**					
Administração pública	49,7	56,8	0,90	1,14	0,79
Entidades empresariais	65,6	60,0	0,53	0,91	0,58
Entidades privadas sem fins lucrativos	59,9	63,5	0,71	1,06	0,68
Média	58,1	59,4	0,67	1,02	0,66
**Norte**					
Administração pública	36,3	50,1	0,96	1,38	070
Entidades empresariais	37,6	20,8	0,35	0,55	0,63
Média	36,7	42,8	0,78	1,17	0,67
**Sudeste**					
Administração pública	57,7	59,0	0,90	1,02	0,88
Entidades empresariais	64,2	59,8	0,69	0,93	0,74
Entidades privadas sem fins lucrativos	55,9	55,6	0,74	1,00	0,74
Média	57,8	57,7	0,81	1,00	0,81
**Sul**					
Administração pública	69,1	66,4	0,71	0,96	0,74
Entidades empresariais	57,2	56,3	0,68	0,99	0,69
Entidades privadas sem fins lucrativos	48,2	46,0	0,71	0,96	0,74
Média	55,8	53,8	0,70	0,96	0,73
**Brasil**					
Administração pública	55,0	58,6	0,88	1,07	0,82
Entidades empresariais	60,3	57,7	0,62	0,96	0,64
Entidades privadas sem fins lucrativos	53,4	52,9	0,73	0,99	0,74
Média	55,6	56,5	0,77	1,01	0,76

Foram identificadas 32 unidades eficientes, referências para os HEs gerais ineficientes (Material Suplementar). Um total de 15 HEs gerais foram eficientes em ambos os anos analisados. Entre as referências, 24 (75,0%) hospitais eram de grande porte, 16 (50,0%) localizados no Sudeste e 11 (34,4%) privados sem fins lucrativos.

A Tabela 4 apresenta a projeção de produção esperada para que todos os HEs gerais ineficientes atinjam a fronteira de melhores práticas a partir de 2021. Num cenário de recuperação, considerando-se a atual disponibilidade de recursos, esses hospitais devem aumentar sua produção em 2.205.856 (96,5%) internações e em 872.264 (107,4%) cirurgias. Paralelamente, com o fim da emergência de saúde global por covid-19, estima-se a queda da TMH de 9,2% para 4,0%, valendo destacar o decréscimo esperado na região Norte (de 11,6% para 3,5%) e entre hospitais privados sem fins lucrativos (de 12,2% para 4,0%).

**Tabela 4:** Projeção esperada para a produção de serviços e a taxa de mortalidade estimada dos hospitais de ensino após a pandemia de covid-19, por região e natureza jurídica, Brasil, 2021

**Classificação dos hospitais de ensino**	**Internações**	**Projeção de internações**	**Diferença (%)**	**Cirurgias**	**Projeção de cirurgias**	**Diferença (%)**	**Mortalidade hospitalar**	**Projeção de mortalidade hospitalar**
**Região/Natureza jurídica**
**Centro-Oeste**								
Administração pública	58.538	145.076	147,8	28.953	65.135	125,0	6,8	2,6
Entidades empresariais	37.648	85.191	126,3	14.311	31.162	117,7	5,2	4,0
Entidades privadas sem fins lucrativos	8.382	21.413	155,5	4.431	11.319	155,5	7,2	3,4
Total	104.568	251.680	140,7	47.695	107.617	125,6	6,2	3,2
**Nordeste**								
Administração pública	145.223	306.072	110,8	74.598	142.563	91,1	7,8	4,5
Entidades empresariais	97.370	200.557	106,0	41.081	88.781	116,1	5,7	4,1
Entidades privadas sem fins lucrativos	48.730	107.156	119,9	30.970	59.748	92,9	8,2	3,8
Total	291.323	613.786	110,7	146.649	291.092	98,5	7,0	4,2
**Norte**								
Administração pública	28.302	81.905	189,4	23.851	48.840	104,8	12,1	3,4
Entidades empresariais	4.237	23.100	445,2	3.123	17.118	448,1	10,0	3,8
Total	32.539	105.005	222,7	26.974	65.958	144,5	11,6	3,5
**Sudeste**								
Administração pública	534.099	1.001.530	87,5	179.058	359.894	101,0	8,3	3,6
Entidades empresariais	143.974	278.643	93,5	46.559	99.817	114,4	7,0	4,6
Entidades privadas sem fins lucrativos	540.095	966.618	79,0	153.543	313.619	104,3	12,7	4,5
Total	1.218.168	2.246.790	84,4	379.160	773.330	104,0	9,9	4,1
**Sul**								
Administração pública	121.340	222.276	83,2	63.573	105.074	65,3	7,8	4,4
Entidades empresariais	237.407	463.996	95,4	52.099	124.809	139,6	6,8	4,2
Entidades privadas sem fins lucrativos	281.572	589.240	109,3	95.732	216.266	125,9	12,8	3,5
Total	640.319	1.275.512	99,2	211.404	446.149	111,0	10,1	3,9
**Brasil**								
Administração pública	887.502	1.756.859	98,0	370.033	721.506	95,0	8,3	3,7
Entidades empresariais	520.636	1.051.486	102,0	157.173	361.687	130,1	6,5	4,2
Entidades sem fins lucrativos	878.779	1.684.427	91,7	284.676	600.953	111,1	12,2	4,0
Total	2.286.917	4.492.773	96,5	811.882	1.684.146	107,4	9,2	4,0

## DISCUSSÃO

Neste estudo, foi observada queda da produtividade dos HEs gerais do Brasil, em todas as regiões e para todas as naturezas jurídicas, no período 2019-2021. A queda de produtividade observada se deveu ao compromisso social dos HEs gerais, e não a um desacerto na política pública. Ou seja, o aumento na oferta de recursos (leitos de CTI) e a queda da produção (internações e cirurgias) se deram para apoiar as ações estratégicas de enfrentamento à pandemia. Da mesma forma, o aumento observado da TMH em vigência da pandemia foi um marcador da frequência de internações por covid-19 nas unidades sob análise, e não um indicador de qualidade da atenção. 

Queda da produção, com menor atenção às demais doenças, foi fenômeno mundia^l.^ 17 Considerada a vocação assistencial dos HEs, responsáveis por 35,3% da produção de alta complexidade no paí^s^,^2^ a queda de produção teve impacto importante na realização de procedimentos mais complexos. Em 2021, os HEs gerais do país reduziram as cirurgias cardíacas em 14,8%, as cirurgias oncológicas em 8,4%, a radioterapia em 96,8% e os transplantes em 18,6^%^.^2^ Estimativas apontavam para 60 mil cirurgias cardiovasculares represadas pela pandemia, aumentando a fila para o procediment^o.^ 18 A queda na produção, em todos os níveis de cuidado, repercutiu na saúde da população brasileira; por exemplo, a mortalidade por doenças cardiovasculares aumentou em 6,9% no mesmo períod^o^.^6^ Acrescentem-se as renúncias relacionadas ao ensino e pesquisa, à exceção das atividades acadêmicas direcionadas à covid-19.

Para a retomada da produtividade, a produção de internações e cirurgias deve praticamente dobrar no país como um todo, e gestores de cada unidade de saúde (assim como de municípios e estados) podem se programar para o quanto precisam incrementar na produção. A ativação dos 4.814 (51,9%) leitos de CTI (maior do que a média nacional, de 46,7%) favorece a vocação de produção da alta complexidade pelos HEs gerais na retomada desses procedimentos. Ou seja, mais recursos de apoio à alta complexidade promovem o incremento desta produção, desde que a unidade se mantenha na fronteira de eficiência.

Como limitação do estudo, vale citar a ausência de modelos qualitativos para a estruturação do problema antes da modelagem matemática. Estud^os19,20,^ 21 sugerem o uso de metodologias associadas (multimetodologia) para avaliação de contextos e preferências antes da escolha dos modelos matemáticos. Com relação às variáveis do modelo, faltaram informações acuradas sobre a contratação de recursos humanos, os quais foram importantes para a produtividade durante a pandemia, e assim poderiam ter entrado no modelo. Dados sobre atividades de ensino e pesquisa também enriqueceriam a análise, uma vez que o volume de pesquisa, alta razão residentes/leitos (intensidade de ensino) e baixa razão residentes/médicos (dedicação de ensino) estão associados ao aumento da eficiênci^a.^ 22 Outra limitação está na heterogeneidade da informação entre as regiões brasileiras. Estudo sobre excesso de óbitos durante a pandem^ia^ 23 sugere maior dificuldade diagnóstica e subnotificação de óbitos por covid-19 em capitais nordestinas se comparadas às capitais do Sudeste. Pesquisa utilizando DEA em redes para estudar capacidade e estruturas de enfrentamento à covid-19 mostrou que as regiões Norte e Nordeste apresentaram maior vulnerabilidade durante a pandemia por falta de estrutura (leitos de CTI) e menor capacidade na realocação de recursos (médicos e respiradores) para o atendimento ao excesso de demanda de pacientes com covid-1^9.^ 24

Como desdobramento, sugere-se a aplicação do modelo DEA-Malmquist nos anos subsequentes ao deste estudo, quando houve introdução da vacina em território nacional (ainda em 2021), além da introdução de ondas sucessivas e novas cepas de covid-19. Para aprimorar o estudo das unidades de referência, pesquisas qualiquantitativas podem identificar padrões de estratégias de enfrentamento exitosas durante a pandemia. A utilização dessa ferramenta, juntamente com uma melhor caracterização da produção desses hospitais, permitirá a estimativa e o monitoramento da recomposição gradativa da oferta de serviços, de acordo com a dinâmica de necessidades e as demandas da sociedade nacional.

## Apêndice

**Tabela Suplementar 1:** Hospitais de ensino gerais eficientes, por região, estado, natureza jurídica e porte, Brasil, 2019 e 2021

Nome do hospital	Região	UF	Natureza jurídica	Porte	Referência (anos)
Fundação Hospital Adriano Jorge	Norte	AM	Administração pública	Grande	2019 e 2021
Hospital Anchieta	Sudeste	SP	Administração pública	Médio	2019 e 2021
Hospital da Baleia	Sudeste	MG	Entidade privada sem fins lucrativos	Médio	2019 e 2021
Hospital da Restauração Governador Paulo Guerra	Nordeste	PE	Administração pública	Grande	2019 e 2021
Hospital das Clínicas da Faculdade de Medicina de Ribeirão Preto	Sudeste	SP	Administração pública	Grande	2019 e 2021
Hospital das Clínicas da Faculdade de Medicina de São Paulo	Sudeste	SP	Administração pública	Grande	2019 e 2021
Hospital das Clínicas da Universidade Federal de Pernambuco (EBSERH^a^)	Nordeste	PE	Entidade empresarial	Grande	2021
Hospital de Base de São José do Rio Preto	Sudeste	SP	Entidade privada sem fins lucrativos	Grande	2021
Hospital de Caridade São Vicente de Paulo	Sudeste	SP	Entidade privada sem fins lucrativos	Grande	2019 e 2021
Hospital do Trabalhador	Sul	PR	Administração pública	Grande	2019 e 2021
Hospital Estadual de Sumaré Dr. Leandro Franceschini	Sudeste	SP	Administração pública	Grande	2021
Hospital Geral do Grajau	Sudeste	SP	Administração pública	Grande	2019
Hospital Getúlio Vargas	Nordeste	PE	Administração pública	Grande	2019 e 2021
Hospital Municipal da Piedade	Sudeste	RJ	Administração pública	Médio	2019 e 2021
Hospital Municipal Universitário de São Bernardo do Campo	Sudeste	SP	Administração pública	Grande	2019 e 2021
Hospital Nossa Senhora do Rócio	Sul	PR	Entidade empresarial	Grande	2019
Hospital Regional do Paranoá	Centro-Oeste	DF	Administração pública	Grande	2021
Hospital Santa Lucinda da Pontifícia Universidade Católica de São Paulo	Sudeste	SP	Entidade privada sem fins lucrativos	Médio	2021
Hospital Universitário Cajuru da Pontifícia Universidade Católica do Paraná	Sul	PR	Entidade privada sem fins lucrativos	Grande	2019 e 2021
Hospital Universitário Clemente de Faria da Universidade Estadual de Montes Claros	Sudeste	MG	Administração pública	Grande	2021
Hospital Universitário da Universidade Federal do Maranhão (EBSERH^a^)	Nordeste	MA	Entidade empresarial	Grande	2021
Hospital Universitário da Universidade Federal de Sergipe (EBSERH^a^)	Nordeste	SE	Entidade empresarial	Médio	2019
Hospital Universitário da Universidade Federal de São Carlos (EBSERH^a^)	Sudeste	SP	Entidade empresarial	Médio	2019 e 2021
Hospital Universitário de Lagarto da Universidade Federal de Sergipe (EBSERH^a^)	Nordeste	SE	Entidade empresarial	Médio	2019
Hospital Universitário Evangélico Mackenzie	Sul	PR	Entidade privada sem fins lucrativos	Grande	2021
Hospital Universitário Júlio Muller da Universidade Federal do Mato Grosso (EBSERH^a^)	Centro-Oeste	MT	Entidade empresarial	Médio	2021
Hospital Universitário Walter Cantídio da Universidade Federal do Ceará (EBSERH^a^)	Nordeste	CE	Entidade empresarial	Grande	2019 e 2021
Irmandade Nossa Senhora das Mercês de Montes Claros	Sudeste	MG	Entidade privada sem fins lucrativos	Grande	2019
Santa Casa de Misericórdia de Fortaleza	Nordeste	CE	Entidade privada sem fins lucrativos	Grande	2019 e 2021
Santa Casa de Misericórdia de Limeira	Sudeste	SP	Entidade privada sem fins lucrativos	Grande	2019
Santa Casa de Misericórdia do Pará	Norte	PA	Entidade privada sem fins lucrativos	Grande	2021
Santa Casa de Misericórdia de Belo Horizonte	Sudeste	MG	Entidade privada sem fins lucrativos	Grande	2021

a) Empresa Brasileira de Serviços Hospitalares.
